# It’s all in your head – how anticipating evaluation affects the processing of emotional trait adjectives

**DOI:** 10.3389/fpsyg.2014.01292

**Published:** 2014-11-11

**Authors:** Sebastian Schindler, Martin Wegrzyn, Inga Steppacher, Johanna Kissler

**Affiliations:** ^1^Department of Psychology, Affective Neuropsychology, University of BielefeldBielefeld, Germany; ^2^Center of Excellence Cognitive Interaction Technology, University of BielefeldBielefeld, Germany

**Keywords:** EEG/ERP, emotion, language, social feedback, feedback anticipation, communicative context

## Abstract

Language has an intrinsically evaluative and communicative function. Words can serve to describe emotional traits and states in others and communicate evaluations. Using electroencephalography (EEG), we investigate how the cerebral processing of emotional trait adjectives is modulated by their perceived communicative sender in anticipation of an evaluation. 16 students were videotaped while they described themselves. They were told that a stranger would evaluate their personality based on this recording by endorsing trait adjectives. In a control condition a computer program supposedly randomly selected the adjectives. Actually, both conditions were random. A larger parietal N1 was found for adjectives in the supposedly human-generated condition. This indicates that more visual attention is allocated to the presented adjectives when putatively interacting with a human. Between 400 and 700 ms a fronto-central main effect of emotion was found. Positive, and in tendency also negative adjectives, led to a larger late positive potential (LPP) compared to neutral adjectives. A centro-parietal interaction in the LPP-window was due to larger LPP amplitudes for negative compared to neutral adjectives within the ‘human sender’ condition. Larger LPP amplitudes are related to stimulus elaboration and memory consolidation. Participants responded more to emotional content particularly when presented in a meaningful ‘human’ context. This was first observed in the early posterior negativity window (210–260 ms). But the significant interaction between sender and emotion reached only trend-level on *post hoc* tests. Our results specify differential effects of even implied communicative partners on emotional language processing. They show that anticipating evaluation by a communicative partner alone is sufficient to increase the relevance of particularly emotional adjectives, given a seemingly realistic interactive setting.

## INTRODUCTION

Language serves many different functions, ranging from the communication of facts and knowledge, to the communication of socio-emotional evaluations. In fact, symbolic interactionism theory suggests, that language meaning is derived from interaction with others (Blumer, 1969). This interaction is supposed to connect the identities of the communicating partners (Burke, 1980). For humans, communication using emotionally relevant language is of special interest (Barrett et al., 2007; Lieberman et al., 2007). Accordingly, newspapers and advertisers often select emotional words for their headlines, as their processing is prioritized (for a review see e.g., Zald, 2003; Kissler et al., 2006; Citron, 2012). However, influence of the social communicative context on emotional word processing has not been addressed elaborately. The present study aims to do so by creating an evaluative context and investigating whether processing of emotion-laden language differs in anticipation of personality evaluation.

So far processing of emotional language has been mostly investigated in the absence of communicative context. Neuroscience research has shown that brain event-related potentials (ERPs) differentiate between emotional and neutral contents during reading (Kissler et al., 2007) and in lexical (Schacht and Sommer, 2009a,b), grammatical (Kissler et al., 2009) or evaluative decision tasks (Naumann et al., 1997). Emotion effects are most consistently reflected in a larger early posterior negativity (EPN) arising from about 200 ms, which is thought to reflect mechanisms of perceptual tagging and early attention (Kissler et al., 2007; Kissler and Herbert, 2013). A more pronounced late parietal positivity (LPP) from about 500 ms after word presentation, has been implicated in elaborative evaluation and memory processing of emotional words (Herbert et al., 2006, 2008; Kissler et al., 2006, 2009; Kanske and Kotz, 2007; Hofmann et al., 2009; Schacht and Sommer, 2009b).

Previous work showed that establishing a self referential context can alter word processing at early (Fields and Kuperberg, 2012), as well as late processing stages (Watson et al., 2007; Shestyuk and Deldin, 2010; Herbert et al., 2011a,b). This implies self-reference as one important source of plasticity in emotion word processing.

According to symbolic interactionism, the discursive context in which emotional language is embedded should likewise be an important source of plasticity in word processing. In social communication, participants have expectations about their communicative partners and react to violations of these expectations (Burgoon et al., 1983, 2000). Therefore, establishing a socially relevant communicative context, rather than solely self-relevance, can be expected to alter the way emotional language is processed.

Receiving feedback from another person regarding one’s own personality represents a highly salient social context. For some people receiving feedback may even pose a social threat, since humans have a strong need to belong to a community (Baumeister and Leary, 1995), seek approval by others (Izuma et al., 2010; Romero-Canyas et al., 2010), and try to avoid unfavorable evaluations (Leary, 1983; Carleton et al., 2011). Electrophysiologically, social threat has been shown to affect early visual ERP components and frontal EEG asymmetry (Crost et al., 2008; Trautmann-Lengsfeld and Herrmann, 2013; Baess and Prinz, 2014). For example, when participants due to group pressure agreed with a wrong answer option, the P1 was reduced compared to a perceptually identical condition (Trautmann-Lengsfeld and Herrmann, 2013). The P1 is one of the first evoked visual potentials. It reflects sensory registration and it is found to be larger for attended stimuli (Mangun and Hillyard, 1991). Influence of social setting is also reported for the N1 (Baess and Prinz, 2014). In a Go/Nogo paradigm, the N1 was found to be larger when both participants had to react in Go trials (Baess and Prinz, 2014). The N1 is thought to be a marker of visual discrimination (Vogel and Luck, 2000) and decreases with repetition (Carretié et al., 2003). Like the P1, the N1 increases when stimuli are attended (Hillyard et al., 1998). P1/N1 modulations have been occasionally reported for emotional stimuli (Pourtois et al., 2004; Keil et al., 2007; Steinberg et al., 2013) and recent evidence shows that also social context may change very early sensory processing.

These electron paramagnetic resonance (EPR) findings are complemented by fMRI results showing a regionally distinct processing of social feedback Social feedback has been shown to activate reward system structures such as the medial prefrontal cortex and the ventral striatum as well as the anterior cingulate cortex, involved in pain processing (Somerville et al., 2006, 2010; Izuma et al., 2008, 2010; Davey et al., 2010; Eisenberger et al., 2011; Korn et al., 2012). Together EEG and fMRI data indicate that effects of social feedback on brain physiology can be observed in artificial laboratory conditions using highly temporally and spatially resolving imaging methods.

As humans constantly make predictions about the future (Koster-Hale and Saxe, 2013; Seth, 2013), even the anticipation of socially relevant feedback, for example delivered as gestural approval or disapproval (‘thumbs up’ or ‘thumbs down’). The present study aims to do so by creating an evaluative context and investigating whether processing of emotion-laden language differs in anticipation of personality evaluation. Produces distinct cerebral activities (Kohls et al., 2013). In this study, the avoidance of social punishment and the anticipation of social reward led to enhanced activity in the ventral striatum and nucleus accumbens (Kohls et al., 2013). This indicates that both the fear of socially unfavorable evaluations and hope of acceptance are central human motives that modulate reward system biology.

The anticipation of socio-emotional language feedback, arguably the most common source of socially relevant feedback, has not yet been investigated. However, there is information on the effects of anticipatory anxiety on ERPs: research demonstrates unspecific sensitizing effects of threat of shock, reflected in more positive-going early ERPs during threat-cue processing (Bublatzky and Schupp, 2012). Trials signaling a possible electric shock, lead to a larger P1 and P2, as well as a larger parietal LPP compared to trials signaling safety (Bublatzky et al., 2010; Bublatzky and Schupp, 2012). Moreover, anticipatory anxiety has been reported to specifically accentuate the processing of emotional pictures, surprisingly leading to a larger EPN for positive pictures when trials are signaling a possible electric shock (Bublatzky et al., 2010). Using anticipation of speaking in public as a threat induction, a different study reported the arguably more intuitive finding of accentuated processing of negative stimuli: participants were told that they would supposedly held a speech in public after completing a face perception task. Compared to a control condition this led to a larger N170 and EPN for angry faces in the face perception task (Wieser et al., 2010).

Anticipation of verbal social feedback likely involves a phase of self-reflection, akin to self-referential processing, perhaps combined with anticipatory anxiety of negative feedback. The intensity of these processes may depend on both the message and the sender of the feedback. Existing studies of emotion word processing have focused on the processing of single words in psycho-linguistic tasks, devoid of social context. However, word meaning will change depending on attributed sender characteristic and direction of communication. In ecologically valid situations, already an inferred psychological context or a psychological attribution to another individual may constitute presence or absence of an interaction. For instance, feedback in the form of the adjective ‘boring’ should be more important if another human is the putative sender rather than a computer. Likewise, ‘boring’ may be regarded as more intense, when it is used to characterize oneself as a person rather than one’s teaching lesson. Similarly, an adjective like ‘cheap’ may be relatively neutral when describing an object, but becomes highly negative when it is used to characterize a person.

Against this background, the present study examines the influence of the putative sender on processing of negative, neutral and positive written adjectives in a social evaluative context. Participants were told that either an unknown other person would evaluate them based on his/her first impression, or a computer program would randomly highlight trait adjectives. In reality, both conditions were random and perceptually identical. We expected that anticipation of feedback by another person would generally change stimulus processing (sensitizing effects, Wieser et al., 2010 or Bublatzky and Schupp, 2012) and investigated whether this occurs at early perceptual (P1, N1), mid-latency (EPN), or late (LPP) processing stages. Moreover, we examined valence-specific interactions between feedback content and evaluative context (human, computer). Generally, in the context of being evaluated by another person, negative, and positive trait adjectives can be expected to induce larger P1, N1, EPN, or LPP amplitudes, reflecting fear of unfavorable evaluations and social rejection (Somerville et al., 2006; Masten et al., 2009; Eisenberger et al., 2011) or hope of acceptance by others (Izuma et al., 2010; Romero-Canyas et al., 2010; Simon et al., 2014).

Against this background, we evaluate the sequence of early (P1, N1), mid-latency (EPN) and late visually evoked potentials in response to adjectives presented as potential trait-feedback by another human or a randomly acting computer.

## MATERIALS AND METHODS

### PARTICIPANTS

Eighteen participants were recruited at the University of Bielefeld. They gave written informed consent according to the Declaration of Helsinki and received 10 Euros for participation. The study was approved by the Ethics Committee of the University of Konstanz. Due to experimentation errors, two datasets had to be excluded, leaving 16 participants for final analysis. The resulting 16 participants (12 females) were 24.40 years on average (SD = 0.66). All participants were native German speakers, had normal or corrected-to-normal visual acuity, and were right-handed. Twelve participants were undergraduate students; four had already received their Bachelor’s or Master’s degree. Screenings with the German version of the Beck Depression Inventory and the State Trait Anxiety Inventory (Spielberger et al., 1999; Hautzinger et al., 2009), revealed no clinically relevant depression (*M* = 4.12; SD = 4.54) or anxiety scores (*M* = 35.94;SD = 3.06).

### STIMULI

Adjectives were previously rated by 20 students in terms of valence and arousal using the Self-Assessment Manikins (Bradley and Lang, 1994). Raters had been specifically instructed to consider adjective valence and arousal in the context of being described by another person with this respective adjective. 150 adjectives (60 negative, 30 neutral, 60 positive) were selected and matched in their linguistic properties, such as word length, frequency, familiarity and regularity (see **Table 1**). Importantly, negative and positive adjectives differed only in their valence. As there is a lack of truly neutral trait adjectives, neutral adjectives were allowed to differ from emotional adjectives on rated concreteness next to valence and arousal.

**Table 1:** Comparisons of negative, neutral and positive adjectives by one-way-anlysis of variances.

Variable	Negative adjectives (*n* = 60)	Neutral adjectives (*n* = 30)	Positive adjectives (*n* = 60)	*F* (2,147)
Valence	3.10^a^ (0.84)	5.01^b^ (0.32)	7.01^c^ (0.90)	371.05***
Arousal	4.57^a^ (0.85)	3.30^b^ (0.66)	4.40^a^ (0.85)	25.93***
Concreteness	3.24^a^ (1.03)	5.07^b^ (1.46)	3.16^a^ (1.27)	28.10***
Word length	8.93 (2.65)	9.23 (2.94)	9.15 (2.48)	0.16
Word frequency (per million)	4.64 (8.56)	4.34 (6.26)	4.78 (8.05)	0.03
Familiarity (absolute)	21805.77 (39221.26)	18832.23 (48387.29)	19331.85 (42795.46)	0.07
Regularity (absolute)	261.58 (551.78)	165.97 (378.73)	239.06 (388.71)	0.44
Neighbors	3.45 (4.44)	2.53 (3.42)	3.78 (4.70)	0.83
Neighbors Levenshtein (absolute	6.13 (6.48)	4.93 (4.14)	6.60 (6.26)	0.76

***p≤ 0.001. Standard deviations appear in parentheses below means; means in the same row sharing the same superscript letter do not differ significantly from one another at *p* ≤ 0.05; means that do not share subscripts differ at *p* ≤ 0.05 based on LSD test *post hoc* comparisons.

### PROCEDURE

Participants were told that they would be rated by an unknown other person or would see ratings generated randomly by a computer program. All subjects underwent both conditions. Sequence was counterbalanced across participants.

Upon arrival, participants were asked to describe themselves in a brief structured interview in front of a camera. They were told that their self-description was videotaped and would be shown to a second participant next door. The interview contained four questions encouraging the participant to talk about their strengths and weaknesses, as well as giving a short biography overview. After the interview, participants filled out a demographic questionnaire as well as BDI and STAI whilst the EEG was applied. To ensure face validity, a research assistant left the testing room a couple of minutes ahead of the fictitious feedback, guiding an ‘unknown person’ to a laboratory room next to the testing room.

**FIGURE 1:**
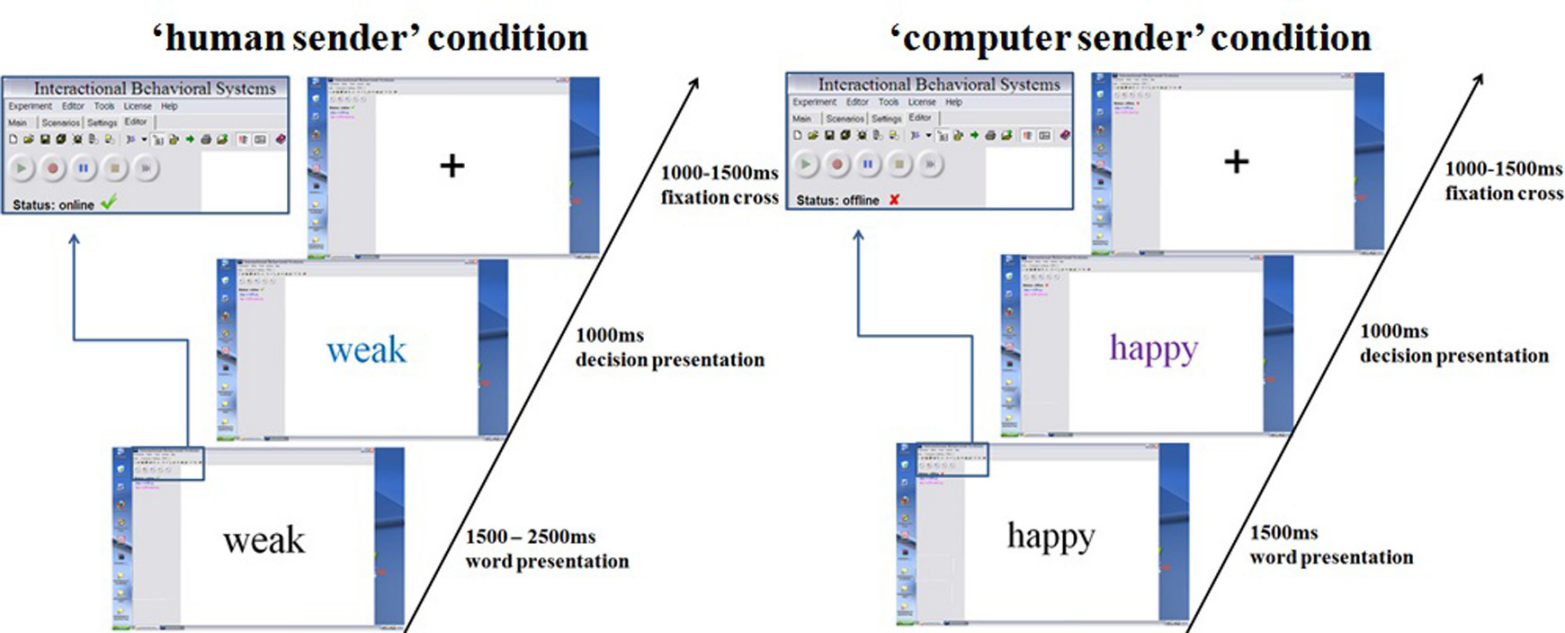
**Trial presentation using the fictitious interactive software.** Each trial started with a presented trait adjective.

Stimuli were presented within a desktop environment of a fictitious program, allegedly allowing instant online communication (see **Figure 1**).

Network cables and changes of the fictitious software desktop image showing a ‘neurobehavioral interactive systems’ environment were implemented to enhance credibility. The 60 negative, 30 neutral, and 60 positive adjectives were randomly presented and feedback upon was randomly generated in both conditions. All adjectives were first presented in black. After a fixed (computer) or variable (human) time interval a color change indicated the feedback on a certain adjective. The presented results relate to the pre-feedback period, when all stimuli still appeared in black. Half of all adjectives were endorsed, leading to 30 affirmative negative, 30 neutral, and 30 affirmative positive decisions. While the presented feedback was randomly generated in both conditions, twenty additionally inserted highly negative adjectives were defined to be always rejected in the ratings to further increase credibility, since it would appear very unlikely for somebody to endorse extremely negative traits in a hardly known stranger. These additional trials were excluded from further analysis. The desktop environment and stimulus presentation were created using presentation^1^. In the ‘human’ condition between 1500 and 2500 ms after adjective onset, color changes indicated a decision by the supposed interaction partner. This manipulation simulated variable decision latencies in humans. The decision was communicated via color change (blue or purple) of the presented adjective, indicating whether the respective adjective applied to the participant or not. Color–feedback assignments were counterbalanced. In the computer condition, corresponding color changes always occurred at 1500 ms, conveying the notion of constant machine computing time. In both conditions color changes lasted for 1000 ms, followed by a fixation cross for 1000–1500 ms. After testing, participants responded to a questionnaire asking them to rate their confidence in truly being judged by another person in the ‘human’ condition, on a five point Likert-scale.

### EEG RECORDING AND ANALYSES

Electroencephalography signals were recorded from 128 BioSemi active electrodes^2^. Four additional electrodes measured horizontal and vertical eye-movement. Recorded sampling rate was 2048 Hz. Pre-processing was done using SPM8 for EEG^3^. Although perhaps best known as a toolbox for the analysis of functional magnetic resonance data, SPM provides a unitary framework for the analysis of neuroscience data acquired with different technologies, including EEG and MEG using the same rationale (Penny and Henson, 2007; Litvak et al., 2011). Oﬄine, data were re-referenced to average reference, downsampled to 250 Hz and butterworth band-pass filtered from 0.166 to 30 Hz. Recorded eye movements were subtracted from EEG data. Filtered data were segmented from 100 ms before word onset until 1000 ms after word presentation. 100 ms preceding word onset were used for baseline-correction. Automatic artifact detection was used for trials exceeding a threshold of 160 μV. Data were averaged, using the robust averaging algorithm of SPM8, excluding possible further artifacts. Overall, less than 1% of all electrodes were interpolated and on average 15.25% of all trials were rejected, leaving on average 50.85 trials for emotional words and 25.43 trials for neutral words for each communicative sender. Artifact rejection rate did not differ between both senders [*F*(1,15) = 0.32, *p* = 0.58], nor between negative, neutral and positive content [*F*(2,30) = 0.26, *p* = 0.78]. There was also no interaction between sender and emotional content regarding artifact rejection rate [*F*(2,30) = 0.09, *p* = 0.91].

### STATISTICAL ANALYSES

Electroencephalography scalp-data were statistically analyzed with EMEGS^4^, (Peyk et al., 2011). Two (sender: human versus computer) by three (emotion: positive, negative, neutral) repeated measure ANOVAs were set-up to investigate main effects of the communicative sender, emotion and their interaction in time windows and electrode clusters of interest. If Mauchly’s Tests of Sphericity yielded significance, degrees of freedom were corrected according to Greenhouse-Geisser as Greenhouse-Geisser ε’s were below 0.75. Partial eta-squared (partial η^2^) was estimated to describe effect sizes, where η^2^ = 0.02 describes a small, η^2^ = 0.13 a medium and η^2^ = 0.26 a large effect (Cohen, 1988). Time windows were segmented from 50 to 100 ms to investigate P1 and from 100 to 150 ms to investigate N1 effects (Bublatzky and Schupp, 2012; Fields and Kuperberg, 2012), from 210 to 260 ms to investigate EPN effects (Kissler et al., 2007) and from 400 to 700 ms to investigate LPP effects (Schupp et al., 2004; Bublatzky and Schupp, 2012).

**FIGURE 2:**
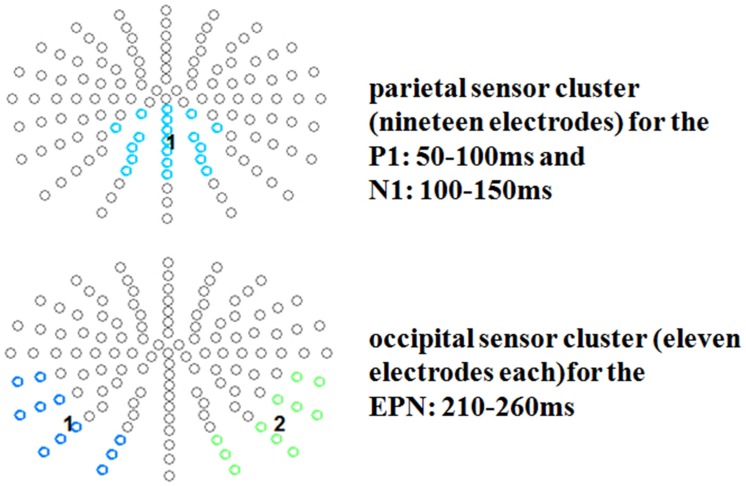
**Selected electrode clusters for the early time windows.** Selected electrodes are highlighted by color.

For the P1 a fronto-central cluster was investigated (13 electrodes: FFC1h, FFCz, FFC2h, FC1h, FCz, FC2h, FCC1h, FCC2h, C1, C1h, Cz, C2h, C2), while for the N1 time window a parietal cluster of nineteen electrodes was examined (CCPz, CP1h, CPz, CP2h, CPP1, CPz, CPP2, P1, Pz, P2, PPO1, PPOz, PPO2, PO1, POz, PO2, POO1, POOz, POO2; see **Figure 2**). For the EPN time window, two symmetrical occipital clusters of eleven electrodes each were examined (left: I1, OI1, O1, PO9, PO9h, PO7, P9, P9h, P7, TP9h, TP7; right: I2, OI2, O2, PO10, PO10h, PO8, P10, P10h, P8, TP10h, TP8).

**FIGURE 3:**
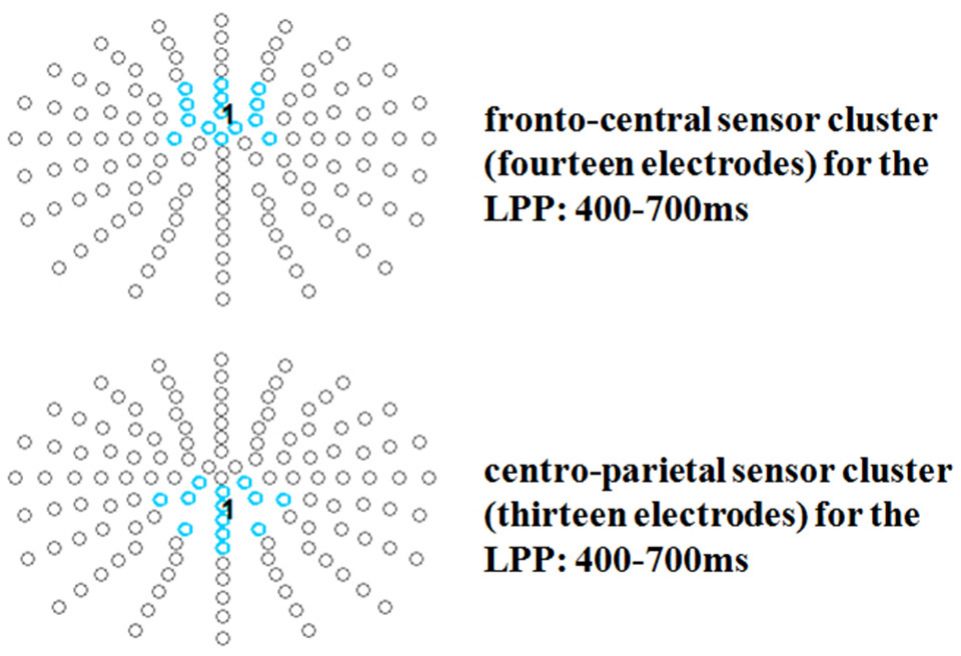
**Selected electrode clusters for the late time window.** Selected electrodes are highlighted by color.

Late positive potential topographies have found to vary, with some authors reporting more parietal others more fronto-central distributions, or even both in one study (Kissler et al., 2009). Since the present data revealed conspicuous differences both at fronto-central and at parietal sites two electrode groups of interest were analyzed for this component. For the LPP time window a fronto-central cluster (14 electrodes: F1h, Fz, F2h, FFC1h, FFCz, FFC2h, FC1h, FCz, FC2h, FCC1h, FCC2h, C1, Cz, C2) and a centro-parietal cluster were investigated (13 electrodes: CCP1h, CCPz, CCP2h, CP1, CP1h, CPz, CP2h, CP2, CPPz, P1, Pz, P2, PPOz; see **Figure 3**).

## RESULTS

### QUESTIONNAIRE DATA

After debriefing, two participants stated that they were strongly convinced that they had been rated by another person in the ‘human’ evaluation condition, six participants said they quite convinced, four participants somewhat convinced, and two participants said they were little convinced. Mean credibility was 3.4 (SD = 1.02) on a Liktert-scale ranging from one to five.

### P1

No significant main effects of sender *F*(1,15) = 0.18, *p* = 0.68, emotion *F*(2,30) = 0.12, *p* = 0.89, partial η^2^ = 0.05 and no interaction *F*(2,30) = 0.52, *p* = 0.59, partial η^2^ = 0.05 was observed over fronto-central regions.

### N1

**FIGURE 4:**
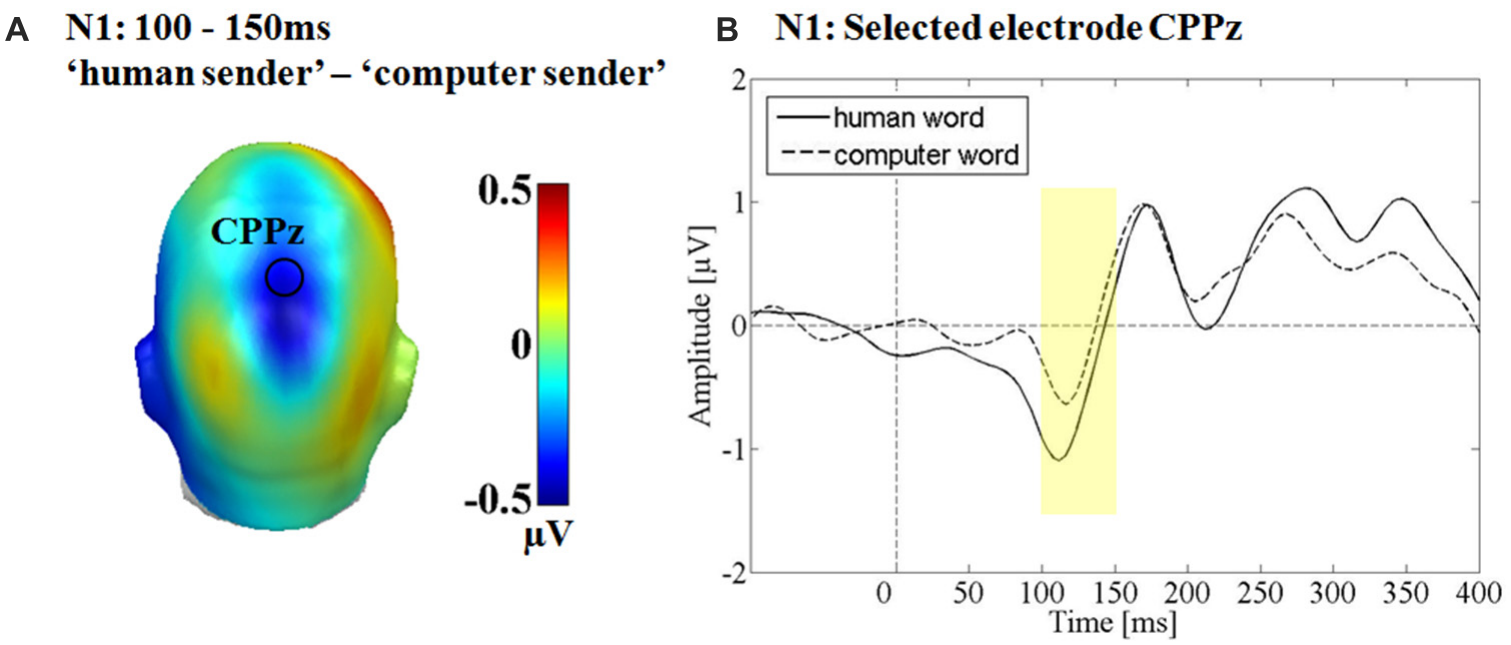
**Results for the main effect of communicative source at the N1. (A)** Difference topographies. Blue color indicates more negativity and red color more positivity in the ‘human sender’ condition. **(B)** Selected electrodes CPPz, displaying the time course over parietal sites.

A significant main effect was observed for the communicative sender over the parietal sensor cluster between 100 and 150 ms *F*(1,15) = 7.51, *p* < 0.05, partial η^2^ = 0.33 (see **Figure 4**). The putative ‘human sender’ evoked a significantly larger N1 compared to the computer sender. There was no main effect of emotion *F*(2,30) = 0.83, *p* = 0.44, partial η^2^ = 0.05 and no interaction between sender and emotion *F*(2,30) = 0.27, *p* = 0.76, partial η^2^ = 0.02.

### EPN

A significant interaction between sender and emotion was observed over occipital sensors during the EPN *F*(2,30) = 3.95, *p* < 0.05, partial η^2^ = 0.21. This interaction was based on a larger EPN for emotional adjectives within the ‘human sender’ compared to a larger EPN for neutral adjectives within the computer sender. However, within the ‘human sender’ *post hoc* comparisons showed only a trend for a larger negativity for positive compared to neutral adjectives (*p* = 0.06) and no differences between negative and neutral words (*p* = 0.55). Within the ‘computer sender’ neutral words elicited a trend- level larger EPN compared to negative words (*p* = 0.08) but not compared to positive words (*p* = 0.28).

There were no main effects of the sender *F*(1,15) = 0.79, *p* = 0.38, partial η^2^ = 0.05 or of the emotional content *F*(2,30) = 0.91, *p* = 0.41, partial η^2^ = 0.06 in the EPN time window.

### LPP

**FIGURE 5:**
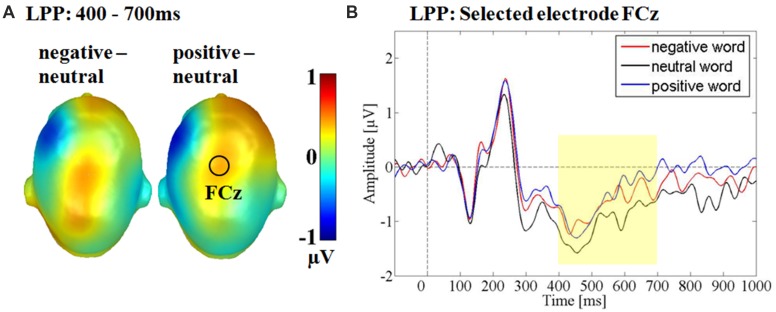
**Main effect for the emotional content in the late positive potential time window. (A)** Head Models for the *post hoc* comparisons within the respective emotion. Blue color indicates more negativity and red color more positivity for the respective difference. **(B)** Selected electrode FCz showing the enhanced positivity for positive and as a trend also for negative adjectives compared to neutral adjectives.

Over the fronto-central electrode cluster, a significant main effect for emotion was observed *F*(2,30) = 3.49, *p* < 0.05, partial η^2^ = 0.19 (see **Figure 5**). *Post hoc* comparisons revealed, that positive adjectives elicited a larger LPP compared to neutral adjectives (*p* < 0.05), while negative compared to neutral adjectives elicited a larger amplitude only in tendency (*p* = 0.13). Positive and negative words did not differ from each other (*p* = 0.59). Over the fronto-central cluster there was no main effect of sender *F*(1,15) = 0.30, *p* = 0.59, partial η^2^ = 0.02 nor an interaction between sender and emotion *F*(1.27,19.11) = 0.20, *p* < 0.83, partial η^2^ = 0.01.

**FIGURE 6:**
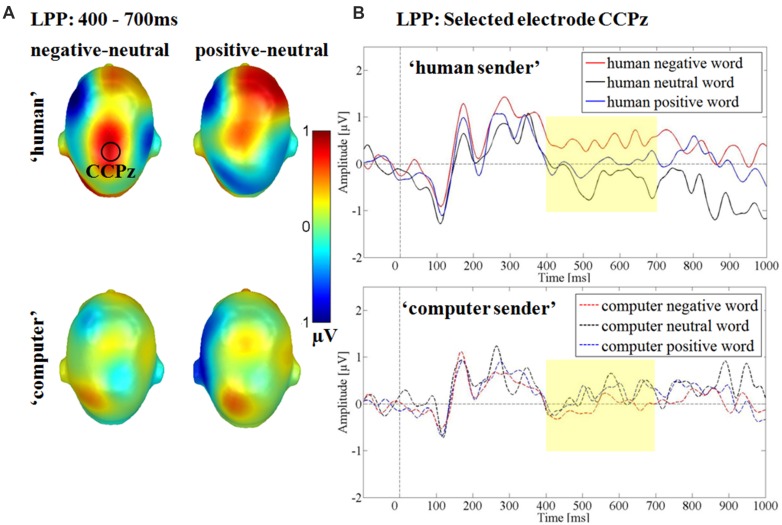
**Interaction between communicative sender and emotional content in the late positive potential time window. (A)** Head Models for the *post hoc* comparisons within the respective communicative sender. Blue color indicates more negativity and red color more positivity for the respective difference. **(B)** Selected electrode CCPz showing the larger positivity for negative compared to neutral adjectives within the ‘human sender’ and small differences between emotional and neutral adjectives within the ‘computer sender.’

Over the centro-parietal electrode group a significant interaction between the communicative sender and emotional content was found *F*(2,30) = 3.46, *p* < 0.05, partial η^2^ = 0.19 (see **Figure 6**). *Post hoc* comparison showed, that within the ‘human sender’ negative words elicited a significantly larger LPP compared to neutral adjectives (*p* < 0.01), while the somewhat larger LPP for positive words compared to neutral words did not reach significance (*p* = 0.15). Negative and positive words did not differ from each other (*p* = 0.17). Within the ‘computer sender’ no differences were found in any comparison (*ps* > 0.49). Over the centro-parietal cluster there were no main effects of sender *F*(1,15) = 0.23, *p* = 0.64, partial η^2^ = 0.02 or emotion *F*(2,30) = 1.31, *p* = 0.29, partial η^2^ = 0.08.

## DISCUSSION

We hypothesized that anticipating an evaluative decision from a human sender would lead to altered processing of trait adjectives by the recipient. A ‘computer sender’ was introduced as a source of random evaluation to provide a maximal contrast between both conditions, while maintaining identical perceptual input. The data reveal effects of sender and emotion as well as interactions. For the ‘human sender,’ a significantly larger N1 between 100 and 150 ms after adjective onset was detected over parietal areas. Starting with the EPN, effects of emotion interacted with perceived sender and in the LPP window, both main effects of sender and emotion as well as their interaction was observed. In the following, we will discuss these findings against the background of the current literature.

An early-onset effect of the ‘human sender’ condition, already in the N1 window, is in line with earlier findings of rapid effects of self-relevance (Fields and Kuperberg, 2012), as well as with sensitizing effects of social threat (Wieser et al., 2010). Within the broader context of the ERP literature, N1 effects suggest more tonic attention orienting toward stimuli supposedly sent by a human. Tonic effects of attention deployment have first been observed by Eason et al. (1969), who also were the first to demonstrate similar effects of volitional attention and threat of an electric shock on visual stimulus processing.

A main effect of emotion was observed in the LPP time window over a fronto-central electrode cluster. Here, positive and in tendency also negative words elicited a larger positivity compared to neutral words. Descriptively, ERPs differed earlier between emotional and neutral adjectives (see **Figure 6**), but interaction effects may have canceled out by stronger main effects of emotion. Brain topographies in the LPP time window differed somewhat between negative and positive adjectives. For the emotion main effect over the fronto-central cluster, a larger positivity was only found for positive adjectives, while for the interaction over the centro-parietal cluster the *post hoc* comparison was only significant for negative adjectives (see **Figures 5** and **6**). LPP topography variations have been found to vary in the same study (Kissler et al., 2009), but not such valence dependent variability. It may be hypothesized that both arousal dependent and valence specific processing, relying on partly differing generator structures exist in the LPP time window regarding positive and negative adjectives.

Processing of positive and negative adjectives was expected to differ between the social evaluation and the feedback condition as reflected in an interaction between emotional content and communicative sender. Early interactions – between 210 and 260 ms – were found over the occipital region. However, *post hoc* comparisons revealed no clearly significant differences within the respective senders. Descriptively, within the ‘human sender’ there was a larger EPN for emotional words, while for the ‘computer sender’ the EPN was somewhat more pronounced for neutral words. Such early (210–260 ms) valence-specific modulations are relatively rare, previous work reported mainly arousal effects in this time window. However, Fields and Kuperberg (2012) reported very early effects of an established self-referential context on word processing. Therefore, it may be specific to the present experimental setting and may be further enhanced by the presently used blocked design.

Between 400 and 700 ms a larger positivity for negative adjectives compared to neutral adjectives was observed over parietal sites within the ‘human sender.’ The comparison between positive and neutral adjectives, while qualitatively similar did not reach significance. For the ‘computer sender’ no differential processing of negative, neutral and positive adjectives could be observed over central sites and in late time windows. The interaction effects indicate that the also reported LPP emotion main effect may be driven partly by the ‘human sender’ (see **Figures 5** and **6**). Such emotion main effects in the LPP time window have been reported previously in typical psycho-linguistic experiments that did not explicitly manipulate context (Herbert et al., 2006, 2008; Kissler et al., 2006, 2009; Kanske and Kotz, 2007; Hofmann et al., 2009; Schacht and Sommer, 2009b). However, as some studies do not find late emotion effects (Rellecke et al., 2011) it may be helpful to consider the communicative context. The present data suggest that emotional differences largely derive from the adopted communicative context or are at least amplified by it. By contrasting a meaningless and a meaningful passive visual word processing condition the differentiation between emotional and neutral words is heightened. Generally, the LPP is associated with elaborative processing and larger LPPs have been shown to predict better subsequent memory (Dolcos and Cabeza, 2002), one might speculate that contextual factors can determine whether emotional material is only transiently attended at early processing stages or elaborated on and commited to memory.

An interaction of emotion with the anticipatory context is in line with findings from shock-threatening (Bublatzky et al., 2010) or from socially threatening situations (Wieser et al., 2010). However, this is the first study which investigated anticipatory effects in a socially relevant communicative context, as extant studies focus on processing of the feedback decision, typically also using fMRI (Somerville et al., 2006, 2010; Izuma et al., 2008, 2010; Davey et al., 2010; Korn et al., 2012). Due to the higher time resolution of the EEG, we were able to investigate how the anticipated feedback on trait adjectives changes in response to the putative sender identity in distinct processing phases. Here, in addition to sensitizing effects due to threat or self-relevance (Bublatzky et al., 2010; Bublatzky and Schupp, 2012; Fields and Kuperberg, 2012) the anticipation of human-generated evaluations led to differential processing of negative adjectives, which was pronounced at later stages. Descriptively, larger differences between emotional and neutral words within the ‘human sender’ compared to the ‘computer sender’ condition could be observed already at the EPN. Emotional words may initially capture more attention resources, but ongoing processing led to a pronounced differentiation between emotional and neutral words, reflected in the enhanced central positivity in the LPP time window for emotional words. As sensitizing effects of threat have previously been found to accentuate selectively positive (Bublatzky et al., 2010) or negative (Wieser et al., 2010) stimulus processing, in this social communicative setting more complex motives may play a role. This could be explained by considerations that humans, in the absence of conflicting evidence, tend to view themselves positively (self-positivity bias), but also fear unfavorable evaluation (Leary, 1983; Somerville et al., 2006; Masten et al., 2009; Carleton et al., 2011; Eisenberger et al., 2011) and seek approval and acceptance by others (Izuma et al., 2010; Romero-Canyas et al., 2010). Perhaps these different motifs play a role at distinct processing stages, maybe even by partly distinct cortical generator structures.

Overall, we cannot exclude that some relevant effects remained undetected, due to the limited number of trials in each cell resulting in limited power. Still, we observed considerable main and interaction effects, suggesting that the study design was able to detect differences between the two putative senders and their effect on processing of emotional trait adjectives during feedback anticipation. Furthermore, credibility ratings for the ‘human sender’ condition indicate successful experimental manipulation of the respective conditions. Self-reported credibility was not significantly correlated with N1 sender differences (two-tailed Pearson correlation *r* = -0.11, *p* = 0.70, *N* = 16; two-tailed Spearman correlation *r*_s_ = -0.31, *p* = 0.25, *N* = 16), making it unlikely that sender main effects could be explained entirely by credibility. A limitation of the presented study may be the generation of adequate neutral trait adjectives. Although all adjectives were tightly matched for all linguistic characteristics, neutral adjectives differed from negative and positive adjectives in arousal and in concreteness. Still, this could neither account for sender differences nor for the valence-specific accentuation of positive or negative contents. Remarkably, the results suggests that in spite of identical perceptual input, the processing of a message, as reflected by electro-cortical activity, changes as a function of the perceived communicative significance. Thus, subjective meaning seems not only to derive from real, but crucially also from supposed interaction with others, connecting not only real but even imaginary identities of communicating partners. In the current study the ‘human sender’ was the only sender able to give meaningful feedback. It would be interesting to compare a putative ‘human sender’ with a ‘computer sender’ able to give personality feedback, to specify unique effects of ‘humanness’ in contrast to only skill attributions. In general this paradigm suggests many different possible sender manipulations which may contribute to our understanding of context influences on (emotional) language processing. Further, it may be worth to know if such very early visual modulations can be replicated in experiments not using blocked within-subject designs.

## CONCLUSION

Summarizing the main results, we found an amplified N1 indicating, regardless of content, the allocation of more early attentional resources to the trait adjectives if the putative sender was another human rather than a randomly operating computer. These differences were present already in anticipation of a decision and using the identical visual input across conditions. In the EPN window, an interaction suggested that emotional adjectives in the human sender condition were processed more intensely, but *post hoc* tests did not reveal clearly significant differences, precluding firm conclusions. Emotional adjectives led to a larger LPP. This interacted with sender: the LPP was particularly large when evaluations were expected from a human sender. This suggests that at early processing stages attention is allocated to all stimuli, indiscriminate of emotional content and only after (or simultaneously with) extraction of content at an evaluative processing stage selective amplification of emotional content in the human sender condition occurs. These findings indicate that imaginary social context has a large impact on language processing within the larger framework of symbolic interactionism.

## AUTHOR CONTRIBUTIONS

Sebastian Schindler and Johanna Kissler contributed to the study design. Sebastian Schindler, Martin Wegrzyn, and Inga Steppacher carried out participant testing, Sebastian Schindler and Johanna Kissler performed statistical analysis, Sebastian Schindler drafted the manuscript under the supervision of Johanna Kissler. Martin Wegrzyn and Inga Steppacher helped to draft and revise the manuscript. All authors read and approved the final manuscript. Sebastian Schindler revised the manuscript under supervision of Johanna Kissler.

## Conflict of Interest Statement

The authors declared that they had no conflict of interest with respect to their authorship or the publication of this article.

## Footnotes

1 http://www.neurobehavioralsystems.com

2 http://www.biosemi.com

3 http://www.fil.ion.ucl.ac.uk/spm/

4 http://www.emegs.org/
